# Sequential Use of Transcriptional Profiling, Expression Quantitative Trait Mapping, and Gene Association Implicates *MMP20* in Human Kidney Aging

**DOI:** 10.1371/journal.pgen.1000685

**Published:** 2009-10-16

**Authors:** Heather E. Wheeler, E. Jeffrey Metter, Toshiko Tanaka, Devin Absher, John Higgins, Jacob M. Zahn, Julie Wilhelmy, Ronald W. Davis, Andrew Singleton, Richard M. Myers, Luigi Ferrucci, Stuart K. Kim

**Affiliations:** 1 Department of Genetics, Stanford University Medical Center, Stanford, California, United States of America; 2 Longitudinal Studies Section, Clinical Research Branch, National Institute on Aging, Baltimore, Maryland, United States of America; 3 Medstar Research Institute, Baltimore, Maryland, United States of America; 4 HudsonAlpha Institute for Biotechnology, Huntsville, Alabama, United States of America; 5 Department of Pathology, Stanford University Medical Center, Stanford, California, United States of America; 6 Stanford Genome Technology Center, Palo Alto, California, United States of America; 7 Laboratory of Neurogenetics, National Institute on Aging, Bethesda, Maryland, United States of America; 8 Department of Developmental Biology, Stanford University Medical Center, Stanford, California, United States of America; Georgia Institute of Technology, United States of America

## Abstract

Kidneys age at different rates, such that some people show little or no effects of aging whereas others show rapid functional decline. We sequentially used transcriptional profiling and expression quantitative trait loci (eQTL) mapping to narrow down which genes to test for association with kidney aging. We first performed whole-genome transcriptional profiling to find 630 genes that change expression with age in the kidney. Using two methods to detect eQTLs, we found 101 of these age-regulated genes contain expression-associated SNPs. We tested the eQTLs for association with kidney aging, measured by glomerular filtration rate (GFR) using combined data from the Baltimore Longitudinal Study of Aging (BLSA) and the InCHIANTI study. We found a SNP association (rs1711437 in *MMP20*) with kidney aging (uncorrected p = 3.6×10^−5^, empirical p = 0.01) that explains 1%–2% of the variance in GFR among individuals. The results of this sequential analysis may provide the first evidence for a gene association with kidney aging in humans.

## Author Summary

Although family studies have shown that genes play a role in longevity, it has proven difficult to identify the specific genetic variants involved. We developed a sequential transcriptional profiling and eQTL mapping approach to find genes associated with aging in the kidney. First, we used genome-wide transcriptional profiling to determine which genes change expression with age in kidney tissue. Next, we used two methods to determine which of these age-regulated genes contain SNPs that associate with expression level. The allele-specific expression method, which compares the mRNA levels of the two alleles within heterozygous individuals, was more sensitive than the total expression method. We tested the eQTLs for association with kidney aging in two populations. One gene that encodes an extracellular matrix protein, *MMP20*, significantly associated with kidney aging, providing the first gene association with kidney aging. Our approach of combining both expression and genotype data can be applied to any phenotype of interest to increase the power to find genetic associations.

## Introduction

Aging trajectories vary among individuals. Both the age at which physiological function begins to decline and the rate of such decline varies among individuals. The heritability of human longevity ranges from 0.23–0.26, but little is known about specific genes that affect the rate of aging or human lifespan [1]. Candidate gene studies have found a few genes in which certain alleles are enriched in centenarians versus the normal population, including *APOC3* (GeneID 345), *IGF1R* (GeneID 3480) and *FOXO3A* (GeneID 2309) [2]–[4]. These alleles may promote better health and contribute toward extended lifespan.

We chose to identify genes that associate with a focused phenotype of aging rather than the nonspecific phenotype of living to age 100. Specifically, we examined aging in the kidney, an organ that shows an objectively quantifiable decline in function with age. With age, the kidney gets smaller, particularly in the cortex, and kidney function begins to measurably decline after age 40–50 [5],[6]. The glomeruli are ball-shaped structures in the kidney composed of capillary blood vessels actively involved in the filtration of the blood to form urine. The rate at which blood is filtered through all of the glomeruli, and thus the measure of the overall renal function, is the glomerular filtration rate (GFR). The major aging phenotype in the kidney is a 25% decline in GFR starting at age 40 [7]. Individuals show variable rates of kidney aging. In one longitudinal study, one third of individuals showed no decrease in GFR measured over a 20 year period, whereas the remainder of the population showed a distinct decline [8]. The heritability of GFR is estimated to be 0.40–0.46 [9],[10]. In a genome-wide association study, single nucleotide polymorphisms (SNPs) in three gene regions (*UMOD*, GeneID 7369; *SHROOM3*, GeneID 57619; *GATM-SPATA5L1*, GeneIDs 2628 and 79029) were shown to associate with GFR [11].

In genome-wide association studies, hundreds of thousands of SNPs are tested, thus the penalty for multiple hypothesis testing is a large obstacle to overcome. A powerful alternative to genome-wide association studies is genomic convergence, which selects candidate genes for a specific phenotype based on genome-wide expression studies [12]–[17]. Differential expression between cases and controls may indicate that the gene is functionally involved in disease pathogenesis. DNA chips can be used to identify gene expression increases or decreases in affected individuals compared to controls, and then SNPs within the genes that change expression can be used as candidates in genetic association studies. This approach scans the entire genome for expression changes associated with a disease in order to prioritize genes with a greater chance of contributing to the disease phenotype. This approach was first used to identify genes associated with Parkinson's disease, schizophrenia, and Alzheimer's disease [12]–[17].

In this study, we have extended the genomic convergence approach to find genes associated with kidney aging by adding an eQTL analysis after the initial genome-wide transcriptional analysis. If a gene is functionally involved in kidney aging and if DNA differences in the gene cause variation in expression among individuals, then there may be an association between the specific allele carried by an individual and that individual's physiological aging trajectory. Finally, we tested the set of eQTLs for association with kidney aging in two studies of normal aging, the Baltimore Longitudinal Study of Aging and the InCHIANTI study. Using this sequential approach, we were able to find SNPs in the matrix metallopeptidase gene *MMP20* (GeneID 9313) that are significantly associated with kidney aging.

## Results

### Selection of Age-Regulated Genes

We used a sequential method involving transcriptional profiling, eQTL mapping and gene association to identify genes that may contribute to kidney aging. We determined which genes change expression with age in the kidney because these are likely enriched for genes that affect physiological aging. For example, a gene that decreases expression with age may contribute to poor renal function because it is expressed at levels below a physiological threshold in the elderly. We obtained a set of 447 age-regulated genes from a genome-wide transcriptional profile of aging in the human kidney [18]. In addition, a previous gene set enrichment analysis identified four genetic pathways that were coordinately age-regulated in each of three human tissues (kidney, muscle and brain). These pathways include 152 extracellular matrix genes, 85 ribosomal genes, 35 chloride transport genes and 95 electron transport chain genes [19]. We combined the age-regulated genes with the age-regulated pathways and obtained a set of 630 genes that change expression with age.

### Identification of eQTLs by Total Expression Analysis

If age-regulated genes are important for kidney function, then variation in gene expression may correlate with kidney function. We focused on finding expression-associated SNPs (eSNPs) using two methods. The first method searched for eQTLs by pooling individuals that have the same genotype for a particular SNP, and then determining whether the different SNP genotypes are associated with average expression of the corresponding gene. We selected 1041 SNPs in the promoter regions and 386 SNPs in the coding and untranslated regions of the 630 age-regulated genes. We then used a custom Illumina GoldenGate assay to genotype these SNPs in 96 kidney samples (Table S1). Total expression data for these 96 samples was obtained from whole-genome microarrays of 69 kidneys from Rodwell et al. (2004) and new expression data from 26 kidney samples. Kidney samples were from normal tissue from patients aged 29 to 92 years. The kidney samples were dissected into cortex (94 samples) and medulla (59 samples). Expression levels of each gene in the genome were determined using Affymetrix HG-U133A and HG-U133B microarrays.

We compared the genotypes from our chosen SNPs to their corresponding gene expression levels and found 16 SNPs in 12 genes associated with total expression level (Linear Regression, p<0.001, Table S2). Four of the genes have two significant SNPs; in two cases, the SNPs are in different linkage disequilibrium blocks indicating that the eSNPs are independent, and in two cases, the SNPs are linked to each other (r^2^>0.8 HapMap CEU population) and thus represent only one significant association [20].

**Figure 1:**
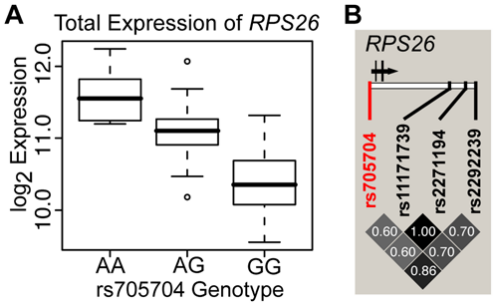
Total expression analysis. Genotypic associations with total expression level. (A) Boxplot of *RPS26* expression according to genotype at the promoter SNP rs705704 (p = 1.2×10^−20^). The boxes define the interquartile range and the thick line is the median. Open dots are possible outliers. (B) Haploview linkage disequilibrium (LD) plot of the *RPS26* region. The SNP rs705704 is 274 bp upstream of the *RPS26* transcription start site. Values in boxes correspond to the pairwise r^2^ LD values (darker boxes correspond to higher r^2^ values) for the HapMap CEU population. rs705704 (red) is partially linked to three SNPs (black) previously shown to associate with *RPS26* expression levels [21]–[25].

One promoter region SNP that showed strong association with total expression is rs705704, which is 274 base pairs upstream of the transcription start site of ribosomal protein S26 (*RPS26*, p = 1.2×10^−20^, Figure 1A). Individuals with the AA genotype have the highest expression, heterozygotes have medium expression, and GG homozygotes have the lowest expression of *RPS26* (GeneID 6231). *RPS26* has been identified as an eQTL in other studies (Figure 1B) [21]–[25].

### Identification of eQTLs by Allele-Specific Expression Analysis

The second method identified differential allelic expression within individuals that are heterozygous for a specific SNP. In this method, the expression levels of each allele are measured directly by assaying SNPs within the mRNA transcript. Heterozygotes were examined for allelic transcript levels that differ from each other, using genomic DNA allelic ratios as a control of 1∶1 hybridization intensity. Because differential expression is examined within heterozygotes, mRNA levels are measured within the same genetic background and cellular environment.

Allele-specific expression analysis was used to test all of the age-regulated genes that had SNPs in their mRNAs. We assayed the relative expression levels of 386 mRNA SNPs in 276 age-regulated genes in 96 individuals. Most of the mRNA SNPs were in the 3′ untranslated regions of genes (249), some were in coding regions (115), and a few were in the 5′ untranslated regions (22).

Oligonucleotides specific for each allele of each SNP were designed for use in the Illumina GoldenGate multiplex PCR assay. Kidney cortex mRNA was reverse transcribed into cDNA prior to the start of the GoldenGate assay. In the assay, the PCR products for each allele were labeled with a different fluorophore and the intensities of each allele were compared to determine if one allele was expressed higher than the other. The cDNA allelic intensities for each SNP were compared within heterozygotes to test for differential allelic expression. Because the intensities from each fluorophore (Cy3 and Cy5) can differ, the genomic DNA allelic intensities of heterozygotes were used as a control to define a 1∶1 allelic ratio for each SNP. The cDNA allelic ratio for each heterozygote was compared to the 95% confidence interval surrounding the mean genomic DNA allele intensity ratio for each SNP. At least five heterozygotes were tested per SNP. If the cDNA allele intensity ratio for more than 50% of individual heterozygotes fell outside the 95% confidence interval and the combined p-value was less than 10^−6^, the SNP was considered to be an eSNP.

**Figure 2:**
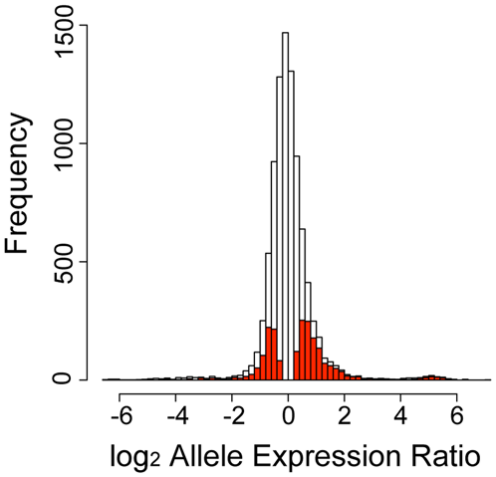
Distribution of allele-specific expression. The white bars show the distribution of the allelic expression ratio for all heterozygotes that express the transcript of the 309 SNPs tested. The red bars show the distribution of the allelic expression ratio for heterozygotes that show allele-specific expression.

In total, 105 eSNPs in 93 age-regulated genes were detected (Table S3, Figure 2). The median fold-change of the higher expressed allele to the lower-expressed allele was 2.1. The level of overexpression of one allele varied widely among genes, from 1.4-fold to apparent monoallelic (>10-fold) expression (Table S3). Two genes (*SPP1*, GeneID 6696 and *TIMP3*, GeneID 7078) had linked eSNPs (r^2^>0.8 HapMap CEU population) that both showed allele-specific differences in expression. Ten genes contained two eSNPs that independently showed differences in expression.

**Figure 3:**
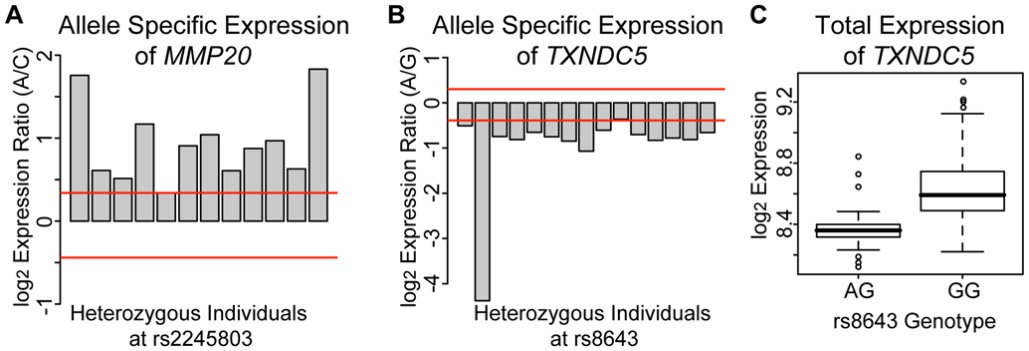
Allele-specific expression analysis. The red lines indicate the 95% confidence interval surrounding the normalized genomic DNA allelic ratio. Each bar represents one heterozygous individual at the particular SNP listed. Individuals above the upper bound or below the lower bound display allele-specific expression. (A) Allele-specific expression was observed at SNP locus rs2245803 in the gene *MMP20* in 11 of 12 heterozygotes tested. The A allele was expressed higher than the C allele in all the individuals displaying allele-specific expression. (B) Allele-specific expression was observed at SNP locus rs8643 in the gene *TXNDC5* in 14 of 15 heterozygotes tested. The G allele was expressed higher than the A allele in all the individuals displaying allele-specific expression. (C) Boxplot of *TXNDC5* total expression according to genotype at the 3′ UTR SNP rs8643 (p = 1.2×10^−4^). The boxes define the interquartile range and the thick line is the median. Open dots are possible outliers.

For most of these eSNPs (96/105), the higher-expressed allele was usually the same across heterozygotes. For example, the A allele is expressed higher than the C allele in 11 of 12 heterozygotes tested at rs2245803 in the gene matrix metalloproteinase 20 (*MMP20*, Figure 3A), and the G allele is expressed higher than the A allele in 14 of 15 heterozygotes tested at rs8643 in *TXNDC5* (GeneID 81567, Figure 3B). In these SNPs, the functional SNP causing the expression difference is likely linked to the SNP we measured. For a smaller subset of the SNPs (9/105 eSNPs), both alleles were observed at a higher level in different heterozygotes. One explanation for this is that the functional SNP causing the expression difference is not closely linked to the SNP we measured in the transcript. Another explanation is that epigenetic effects such as imprinting could cause the differences in expression from the two homologs. For example, one of the genes in which either allele was associated with higher expression is *PEG3* (GeneID 5178, *paternally expressed 3*), which is known to be imprinted [26],[27]. Presumably, the higher-expressed allele in our studies is from the paternal homolog.

386 SNPs were tested for association with expression by both the allele-specific method and the total expression method. While 105 eSNPs were identified by the allele-specific method, only five eSNPs were identified by the total expression method. Of the five SNPs found by the total expression method, four were also found by the allele-specific expression method (Bold in Table S3). One example is rs8643 in the gene *TXNDC5*, in which both methods found that the G allele is associated with higher expression than the A allele (Figure 3B and 3C). These results indicate that the allele-specific assay identified many more eSNPs and is likely more sensitive in detecting expression differences than the total expression assay. A probable reason is that for the allele specific assay, expression is measured from two alleles in heterozygotes and thus variability due to genetic background and environmental effects are reduced or eliminated.

### Genetic Association with Kidney Aging

Our sequential experimental approach identified 101 genes that show age-related changes in expression in the kidney and that also contain eSNPs, indicating a presence of functional polymorphisms. We used these eQTLs as candidates in a gene association study of normal kidney aging. We genotyped a total of 2038 SNPs within these 101 genes (Table S4) in two different cohorts selected to study normal aging. In these studies, the function of the kidney was measured by GFR using 24-hour creatinine clearance. The first cohort is the Baltimore Longitudinal Study of Aging (BLSA), which is a long-running study of human aging begun in 1958 [28]. This study has enlisted over 3000 healthy volunteers from the Baltimore area for clinical evaluations of many age-related traits and diseases [29]. GFR was measured at multiple ages for each individual, with an average of 3–4 measurements per individual taken at different times spanning decades. Thus, this study shows not only the average level of kidney function with respect to age, but also shows the age-related downward trend in kidney function for each individual. Multiple GFR measurements and genotype data were available for 1066 participants.

The second cohort is the InCHIANTI study, which is a population-based epidemiological study aimed at measuring factors important for aging in the older population living in the Chianti region of Tuscany, Italy [30]. About 90% of the elderly from two towns participated in this study, making it an exceptionally useful source to study genetic determinants of normal aging. GFR measurements were performed at one age in 1130 individuals. Characteristics of both cohorts are shown in Table 1.

**Table 1:** Characteristics of kidney aging study samples.

	BLSA	InCHIANTI
	Mean (SD) or *n*	Mean (SD) or *n*
Age	57.6 (17.1)	68.4 (15.5)
Date of Birth	1932 (13.5)	1931 (15.5)
No. Subjects	1066	1130
No. GFR measurements per subject	3.4 (2.6)	1 (0)
No. Male datapoints	2313	515
No. Female datapoints	1359	615
24-hour Creatinine Clearance	112.9 (42.4)	82.4 (30.2)

We used regression models that included age as a covariate to test the SNP genotypes in each population for association with GFR (See Methods). In order for an allelic association with GFR to be considered significant, we first required evidence of association in both populations (p<0.05 in each population). A total of 13 genes contained SNPs that met these criteria (Table 2). Next, we combined these p-values using Fisher's meta analysis, a method for combining p-values from independent tests with the same overall hypothesis [31]. To correct for multiple hypothesis testing, we performed 1000 permutations of each model by swapping identification labels and keeping the genotypes together to preserve linkage disequilibrium (See Methods). Two linked SNPs (rs1711437 and rs1784418) in matrix metalloproteinase 20 (*MMP20*) remained significant after permutation testing (uncorrected p<5×10^−5^, corrected p = 0.01).

**Table 2:** Top SNPs that show association with kidney aging in two populations.

Gene	SNP	Model	BLSA P	InCHIANTI P	Fisher's Meta P*	Permuted P
*MMP20*	rs1711437	DOM	0.0017	0.0015	3.6×10^−5^	1.0×10^−2^
*IGF1R*	rs11630259	REC	0.0001	0.0443	7.8×10^−5^	NS
*RGS6*	rs8007684	ADD×AGE	0.0165	0.0009	1.9×10^−4^	NS
*FAM83F*	rs3021274	DOM×AGE	0.0063	0.0234	1.4×10^−3^	NS
*MMP25*	rs1004792	REC×AGE	0.0038	0.0427	1.6×10^−3^	NS
*ADCY1*	rs11766192	REC×AGE	0.0352	0.0054	1.8×10^−3^	NS
*ADAMTS5*	rs10482979	REC	0.0169	0.0211	3.2×10^−3^	NS
*GPC5*	rs342693	REC×AGE	0.0325	0.0149	4.2×10^−3^	NS
*MTR*	rs2275568	ADD	0.0286	0.0319	7.3×10^−3^	NS
*RPL15*	rs2360610	DOM	0.0469	0.0226	8.3×10^−3^	NS
*GLRB*	rs17035648	DOM×AGE	0.0252	0.0474	9.2×10^−3^	NS
*GPC6*	rs4612931	DOM×AGE	0.0496	0.0270	1.0×10^−2^	NS
*SOHLH2*	rs9593921	DOM×AGE	0.0380	0.0419	1.2×10^−2^	NS

*: Calculated only if individual p-values from each population were <0.05.

We considered whether associations found in the BLSA cohort could have been due to population structure. Concern for population structure was minimal in the InCHIANTI cohort because it is a homogeneous Italian population. Most of the BLSA cohort is made of Caucasian individuals (84%). Our mixed-effect regression model included a covariate for self-reported race, which should control for differences due to population structure. In addition, we found that rs1711437 in *MMP20* showed an association with kidney aging using only data from self-reported Caucasians in the BLSA cohort (uncorrected p = 0.0010). These results indicate that the *MMP20* SNPs associate with kidney aging *per se*, and are not artifacts arising from genetic differences between races.

A SNP in the insulin-like growth factor 1 receptor gene (*IGF1R*) was strongly associated with GFR when taking age into account in the meta-analysis (rs11630259, p = 7.8×10^−5^, Table 2). Decreased activity of this gene has been associated with longer lifespan in model organisms and humans [3],[32],[33]. However, SNPs in *IGF1R* did not remain significant following permutation testing. Therefore, further studies are required to establish a connection between this SNP and kidney aging.

**Figure 4:**
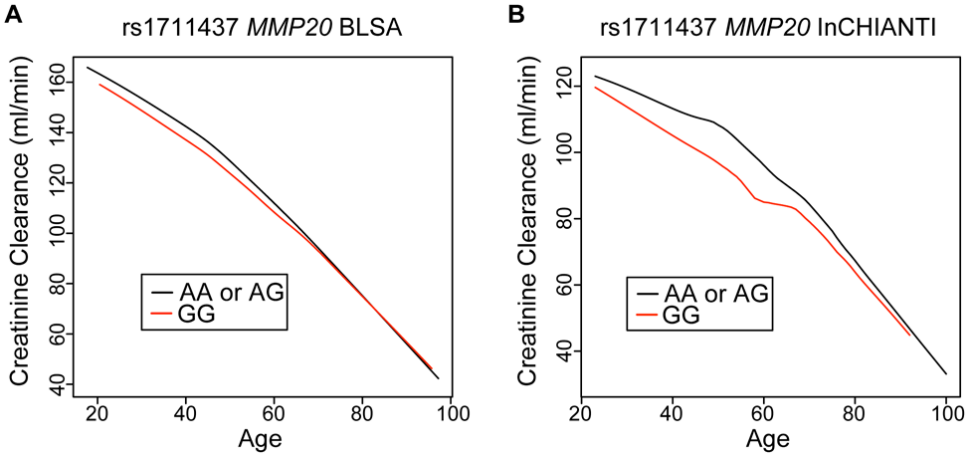
A SNP in *MMP20* associates with a kidney aging phenotype. Loess smoothing lines through a scatter plot of creatinine clearance versus age stratified by genotype at rs1711437 in the BLSA (A) and InCHIANTI (B) populations. (corrected p = 0.01).

In both populations, one or two copies of the A allele at rs1711437 in *MMP20* associated with a higher GFR (Figure 4). For an individual who carries the A allele, his or her creatinine clearance is approximately that of someone 4–5 years younger who does not carry the A allele. In the BLSA population, the genotype of rs1711437 explains 2.1% of the variation in creatinine clearance and in the InCHIANTI population, the genotype explains 0.9% of the variation. Similar results were found for the second SNP rs1784418, which is in linkage disequilibrium with rs1711437.

**Figure 5:**
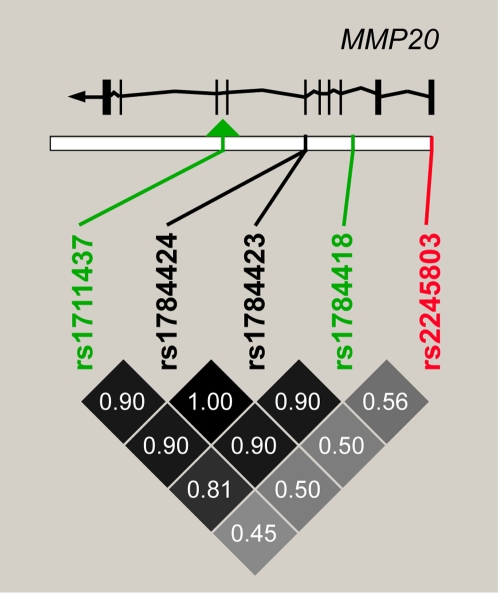
Linkage disequilibrium pattern of *MMP20*. The two SNPs (green) for which we found significant associations with kidney aging are located in introns of *MMP20*. They are linked to each other and to two nonsynonymous SNPs (black) located in exon 6 of *MMP20*. Pairwise r^2^ LD values (darker boxes correspond to higher r^2^ values) from the HapMap CEU population are displayed. These four SNPs are not linked to the SNP (red) in exon 1 that associated with expression level of the gene.

Both rs1711437 and rs1784418 are associated with variation in kidney aging, but the functional SNP is not known. The eSNP rs2245803 identified by allele-specific expression analysis is not linked to rs1711437 and rs1784418 (Figure 5). Thus, some other SNP in this linkage disequilibrium block, such as a coding SNP or a different eSNP, may cause differences in activity of *MMP20* and be responsible for association with the kidney aging phenotype. Interestingly, two nonsynonymous coding SNPs, rs1784424 (Asn281Thr) and rs1784423 (Ala275Val) are contained within this linkage disequilibrium block (Figure 5). These amino acid differences might affect MMP20 function and these coding changes may be causal for differences in kidney aging among individuals.

## Discussion

The goal of our approach was to converge on genes that influence human kidney aging through sequential genomic analyses. We began with a genome-wide transcriptional profile of aging in the human kidney, which gave an unbiased view of gene expression changes that occur with age [18]. Then, we used total expression analysis and allele-specific expression analysis to determine which alleles are differentially expressed. We identified 101 age-regulated eQTLs. SNPs in one of these genes, *MMP20*, showed a statistically significant association with normal kidney aging. Although significant by combining the data from two independent populations, the best way to confirm our gene association with renal aging is to replicate the findings in additional populations.

The populations used to identify aging SNPs, BLSA and InCHIANTI, stand out for their usefulness in studying normal kidney aging. Both of these studies were purposefully designed to study healthy individuals, instead of those harboring diseases associated with old age. The BLSA study includes longitudinal measurements of traits associated with normal aging, which added considerable power to the analysis.

Two SNPs in *MMP20* significantly associated with age-related decline in GFR of the kidney. Matrix metalloproteinases are involved in the breakdown of extracellular matrix in normal physiological processes, such as embryonic development, reproduction, and tissue remodeling, as well as in disease processes, such as arthritis and metastasis [34],[35]. Matrix metalloproteinases degrade extracellular matrix proteins including laminin, elastin, proteoglycans, fibronectin, and collagens [36]. A role for *MMP20* in renal function has not been described before, although previous studies show that *MMP20* plays an important role in tooth development [37]. The finding that a matrix metalloproteinase is involved in kidney aging is striking because changes in the extracellular matrix play a key role in aging of the kidney. The glomerular basement membrane thickens, and the mesangial matrix increases in volume with age [38]. Interstitial fibrosis occurs during aging because of an increase in matrix and fibrillar collagen accumulation in the subintimal space [39].

*MMP20* was included in our candidate aging gene set not because the gene itself is significantly age-regulated in the kidney. Instead, *MMP20* was included because it is a component of the extracellular matrix, one of the pathways that coordinately increased expression with age in three human tissues including the kidney [19]. Therefore, polymorphisms in *MMP20* may not only associate with aging of the kidney, but may associate with phenotypes of aging in other tissues as well. Additionally, if *MMP20* is a common regulator of aging, certain alleles may also be enriched in centenarians.

The second-highest scoring gene in our kidney aging association study is the insulin-like growth factor 1 receptor. Although the SNP in this gene did not reach statistical significance in this study, this result is interesting because this gene is part of the insulin-like signaling pathway that has been shown in be involved in aging in worms, flies and mice [40]. Specifically, reduced signaling in this pathway results in longer lifespans for these model organisms. In worms, the orthologous gene is called *daf-2* (GeneID 175410), and *daf-2* mutants can have lifespans that are 100% longer than wild-type worms [33]. In humans, rare variants in the *IGF1R* gene in centenarians are associated with reduced IGF1R levels and defective IGF signaling [3].

Sequential use of transcriptional profiling and eQTL mapping could be used as a general method to increase the statistical power for any human gene association study. Like candidate gene approaches, an advantage of our approach to identify variants associated with kidney aging is that it increases the statistical power of the gene association study by decreasing the number of SNPs that are tested to potentially functional SNPs. An advantage of our sequential approach over a candidate gene approach is that the entire genome was screened for genes that are age-regulated in the first step.

Several groups have used DNA microarrays to measure gene expression in lymphoblastoid cell lines and have found polymorphisms that associate with expression level [21], [23], [41]–[47]. In a total expression analysis of human brain cortical tissue, 21% of genes have SNPs that associate with expression levels [22]. Other groups have used the allele-specific expression approach to identify differentially-expressed genes in lymphoblastoid cell lines [48]–[51], brain [52], white blood cells [53], fetal kidney and fetal liver [54]. These studies found that 20–50% of the genes in the genome are differentially expressed. Sixteen of the genes showing allele-specific expression found by our study were also found in previous studies (Table S5) [50], [53]–[55]. Thus, 77 of the 93 allele-specifically expressed genes identified in this work represent novel findings. Our finding that 41% of tested genes showed allele-specific expression is similar to the percentage found in previous studies [48]–[54].

Of the expression-associated SNPs we identified, most were found using allele-specific expression measurements within heterozygotes. Specifically, 41% of genes assayed contained eSNPs using the allele-specific expression method, whereas only 2% of genes assayed contained eSNPs using the total expression method. The statistical cutoff for finding eSNPs using the allele-specific method was more stringent than the one used for the total expression method. Thus, our results may underestimate the improved sensitivity of the allele-specific method over the total expression method.

Unlike the total expression method, the allele-specific method examines alleles within the same cellular environment in heterozygous individuals. This maximizes the sensitivity of the assay because the alleles are expressed from the same environment and genetic background. Previous work with a smaller set of 64 genes also showed that allele-specific analysis in heterozygotes was more sensitive than total expression methods for finding SNPs associated with expression levels in *cis* [48]. The results from the allele-specific analysis demonstrate that differential expression is widespread across the human genome and suggest that differential expression could be a major factor contributing to differences in phenotype among individuals. As the Genotype-Tissue Expression (GTEx) project [56] moves forward, it will be important to consider allele-specific expression data to maximize sensitivity to detect differential expression.

Finding new human aging genes, possibly *MMP20*, contributes to our understanding of molecular mechanisms underlying the human aging process. Among young individuals, an unfavorable SNP genotype may indicate risk for rapid decline in kidney function and this information could be extremely useful to identify patients who may require early intervention. Among older individuals, a favorable SNP genotype may indicate that they may still be eligible as kidney donors even though they are over the current upper age limit. As more aging genes are confirmed, the alleles belonging to a patient can be combined to better predict the aging trajectory of the kidney.

## Methods

### Ethics Statement

Ethical approval for the study was obtained from the Stanford University Institutional Review Board (IRB). All subjects provided written informed consent for the collection of samples and subsequent analysis. This study was conducted according to the principles expressed in the Declaration of Helsinki.

### Stanford Kidney Samples

Normal kidney tissue was obtained from Stanford University Medical Center with informed consent either from biopsies of kidneys from transplantation donors or from nephrectomy patients with localized pathology. Kidney tissue from nephrectomy patients was harvested meticulously with the intention of gathering normal tissue uninvolved by the tumor. Samples that showed evidence of pathological involvement or in which there was only tissue in close proximity to the tumor were not used. Kidney sections were immediately frozen on dry ice and stored at −80°C until use.

### RNA and DNA Preparation

Frozen kidney samples were weighed (25–50 mg), cut into small pieces on dry ice, and then placed in 1 ml of TRIzol Reagent (Invitrogen, Carlsbad, California, United States) for RNA extraction or 600 µl of Buffer RLT Plus (Qiagen, Valencia, California, United States) for DNA extraction. The tissue was homogenized using a PowerGen700 homogenizer (Fisher Scientific, Pittsburgh, Pennsylvania, United States). Total RNA was isolated according to the TRIzol Reagent protocol and genomic DNA was isolated according to the Qiagen AllPrep DNA/RNA Mini Kit protocol.

### SNP Selection

Candidate aging genes were chosen from previous transcriptional profiling studies and include 447 age-regulated kidney genes [18] as well as the genes in the four pathways that are commonly age-regulated in the kidney, muscle and brain: extracellular matrix, ribosome, chloride transport and electron transport chain [19]. The candidate kidney aging genes were first searched for mRNA SNPs that could be used in an allele-specific expression assay. In addition to being within the transcript on an autosome, the SNPs had to have a minor allele frequency greater than 0.05 in the HapMap CEU population, an Illumina SNP score greater than 0.4, and be greater than 30 bp from an exon boundary (NCBI Build 36.1) to ensure the Illumina genotyping assay would work properly for both genomic DNA and cDNA. For genes that had multiple assayable mRNA SNPs, those closest to the 5′ end of the gene were chosen, with a maximum of two SNPs per gene. These criteria were met for 386 SNPs in 276 genes. For candidate aging genes that did not have an appropriate mRNA SNP, promoter region (defined as 5 kb upstream or downstream of the transcription start site) SNPs meeting the same minor allele frequency (>0.05) and SNP score (>0.4) criteria were chosen. One to four SNPs were chosen per gene for analysis, totaling 1041 promoter SNPs in 354 candidate aging genes.

### Genotyping

The candidate aging SNPs were genotyped using a GoldenGate Custom Panel from Illumina (San Diego, California, United States). Oligonucleotides specific for each allele of each SNP were designed for use in a multiplex PCR. A standard protocol designed by Illumina and implemented at the Stanford Human Genome Center was used to determine the genotypes of the 96 individuals for whom we had kidney tissue. Samples were hybridized to custom Sentrix Array Matrices and scanned on the Illumina BeadStation 500GX. Allele calls were determined using the Illumina BeadStudio clustering software. The genotyping was successful (>90% call rate, HWE p>0.001) at 1341/1427 of the SNP loci in 599/630 genes (95%). The 1341 SNPs are listed in Table S1.

### Total Expression Quantification

Most of the microarrays (68 cortex and 59 medulla samples) used in our total expression association study were previously analyzed [18]. The same Affymetrix (Santa Clara, California, United States) HG-U133A and HG-U133B high-density oligonucleotide arrays used in Rodwell et al. were used here to measure total expression levels in 26 additional cortex samples. The samples were processed at the Stanford Genome Technology Center using their standard protocol [18]. Eight micrograms of total RNA was used to synthesize cRNA for each sample, and 15 µg of cRNA was hybridized to each microarray. Using the dChip program [57], microarray data (.cel files) were normalized according to the stable invariant set, and gene expression values were calculated using a perfect match model. All arrays passed the quality controls set by dChip. The raw microarray data are available at the Stanford Microarray Database (http://smd.stanford.edu).

### Ancestry Analysis

Because our kidney tissue samples were from individuals living in the diverse San Francisco Bay Area, we needed to control for population structure. Most of the individuals in our study self reported their ancestry (84/96). Genetic clustering analysis has been shown to highly correlate with self-identified ancestry [58]. To determine the ancestry of the 12 unknown individuals, we used the clustering program STRUCTURE [59]. We used the genotypes of 839 unlinked SNPs from our 96 samples and from the CEU, YRI, and JPT+CHB HapMap populations in our analysis. Using the STRUCTURE admixture model, we determined our Stanford samples cluster with the greatest probability into three populations, each clustering with one of the HapMap populations. Because most of the Stanford samples were predominantly of Caucasian genetic ancestry and because it is simplest to use a Boolean covariate value in regression analysis when chronological significance of the state (genetic ancestry in this case) is unknown, we chose to divide the individuals into two groups. In the first group we included individuals with an average percent CEU ancestry >75%. This group included 72 individuals. The second group contained the other 24 individuals. The 84 self-reported ancestries matched the ancestries calculated with STRUCTURE.

### Total Expression Regression Models

We used a linear regression model to determine which SNP genotypes showed a statistically significant association with gene total expression levels:
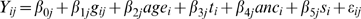
(1)In equation 1, *Y_ij_* is the base 2 logarithm of the expression level for the gene of SNP *j* in kidney sample *i*, *g_ij_* is the genotype (0,1,2 for AA, AB, BB) of individual *i* at SNP *j*, *age_i_* is the age in years of the individual *i*, *t_i_* is 0 if sample *i* was from kidney cortex and 1 if sample *i* was from kidney medulla, *anc_i_* is 0 if the individual contributing sample *i* has >75% CEU ancestry and 1 for other ancestry proportions, *s_i_* is 0 for males and 1 for females, and *ε_ij_* is a random error term. The coefficients *β_kj_* for *k* = 0–5 were estimated by least squares from the data. Our primary interest was *β*_1*j*_ values that significantly differed from zero, indicating that SNP *j* associates with total expression level. Because our microarrays were processed on two different scanners three years apart, we analyzed the two sets of data separately. The first set comprised the 127 samples previously analyzed in Rodwell et al. and the second set comprised the 26 additional samples processed here. We combined the results from the two regression analyses using Fisher's combined probability test [31]. The *β*_1*j*_ p-values from each of the two analyses were combined into one test statistic (χ^2^) having a chi-square distribution and four degrees of freedom using the formula:
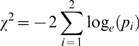
(2)Using Fisher's method, we found 11 promoter SNPs in seven genes and five mRNA SNPs in five genes that associated with total expression level (p<0.001).

### Allele-Specific Expression Quantification

Total RNA was reverse transcribed into cDNA using the SuperScript Double-Stranded cDNA Synthesis Kit (Invitrogen, Carlsbad, California, United States). The same Illumina GoldenGate Custom Panel used for genotyping was used to measure cDNA levels according to which allele of the SNP is present in the transcript. Only SNPs for which the DNA genotyping was successful were analyzed. After the cDNA PCR products were hybridized and scanned, the raw allelic intensities were first used to determine which transcripts were expressed. The expression threshold was defined by the absent allele in normal homozygotes. That is, for an AA genotype, the intensity of the B allele was taken to be background. The expression threshold was calculated for each SNP as the mean of the background intensity plus two standard deviations. SNPs with five or more heterozygotes showing expression of at least one of the two alleles were carried through the rest of the analysis. Of the SNPs measured, 309 of them in 225 genes were genotyped correctly (call rate>90%, HWE p>0.001) and expressed above a background threshold in at least 5 heterozygotes. To determine which alleles were associated with expression level, a confidence interval was calculated for each SNP using the DNA allele intensities of heterozygotes. The confidence interval for each SNP was defined as the mean of the normalized DNA allele A/B raw intensity ratios plus or minus two standard deviations. If the cDNA allele intensity ratio for more than 50% of individual heterozygotes fell outside the 95% confidence interval and the meta p-value [31] was less than 10^−6^, the SNP was considered to be an eSNP. eSNPs were not observed simply due to low, noisy transcript levels because the relative abundance of each gene in the total cDNA sample (calculated from whole-genome microarray data) was greater than the relative abundance of the gene in the genomic DNA sample.

### BLSA Samples

The Baltimore Longitudinal Study of Aging (BLSA) is an intramural research program within the National Institute on Aging [28]. Healthy volunteers aged 18 and older were enrolled in the study starting in 1958. BLSA participants are predominantly Caucasian, community-residing volunteers who tend to be well-educated, with above-average income and access to medical care. These subjects visit the Gerontology Research Center at regular intervals for two days of medical, physiological, and psychological testing. Each participant has a health evaluation by a health provider (physician, nurse practitioner, or physician assistant). Currently, the study population has 1450 active participants, aged 18–97 years (http://www.grc.nia.nih.gov/branches/blsa/blsa.htm). The level of kidney function in the participants has been measured longitudinally in each individual between 1 and 16 times over a 10 to 50 year time period. The kidney aging phenotype of glomerular filtration rate (GFR) was measured by calculating creatinine clearance. Specifically, serum creatinine and 24-hour urinary creatinine levels were obtained from participants using standard clinical procedures [60], and were used to calculate creatinine clearance as follows:
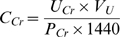
(3)where *C_Cr_* is creatinine clearance in ml/min, *U_Cr_* is urinary creatinine concentration, *V_U_* is the volume of urine collected over 24 hours, *P_Cr_* is the plasma concentration of creatinine, and 1440 is the number of minutes in 24 hours. We were granted access to genotype and GFR data for 1066 individuals. The genotype data comprised the 2038 SNPs genotyped on the Illumina HumanHap550 Genotyping BeadChip that are within the 101 genes that contain SNP associations with expression and have minor allele frequencies >0.01 (Table S4). The GFR data included 3672 creatinine clearance measurements.

### InCHIANTI Samples

The participants in the InCHIANTI study consist of residents of two small towns in Tuscany, Italy [30]. The study includes 1320 participants (age range 20–102 yrs), who were randomly selected from the population registry of Greve in Chianti (population 11,709) and Bagno a Ripoli (population 4,704) starting in 1998 [30]. Over 90% of the population that were over the age of 65 participated in this study, and thus the cohort is a good representation of normal aging (http://www.inchiantistudy.net).

GFR was calculated using creatinine clearance from 24-hour urine collection as in the BLSA study. In this study, the measurement for creatinine clearance was performed at one age only. The genotype data generated by HumanHap550 Genotyping BeadChip consisted of the same 2038 SNPs in 101 candidate aging genes obtained from the BLSA (Table S4). The sample size was 1130 individuals.

### Glomerular Filtration Rate Regression Models

Due to the longitudinal nature of the BLSA data, we used a mixed-effect regression analysis to search for SNP associations with creatinine clearance. Because the creatinine clearance measurements within one subject over time are correlated, the regression coefficients are allowed to vary between individuals. First, we developed the following model using a likelihood ratio approach to explain how creatinine clearance changes with time:
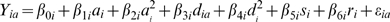
(4)In equation 4, *Y_ia_* is the creatinine clearance of subject *i* at age *a, a_i_* is the age of subject *i*, *d_ia_* is the date in decimal years of the visit of subject *i* at age *a*, *s_i_* is the sex of subject *i*, *r_i_* is the self-reported race of subject *i*, and *ε_ia_* is a random error term. Most of the data points (84%) came from self-reported Caucasian individuals. These individuals were coded 0 for the *r_i_* term and everyone else was coded 1. The coefficients *β_ki_* of each subject *i* for *k* = 0–6 were estimated by maximum likelihood from the data using the “lmer” function from the “lme4” package of R version 2.8.0. Next, to determine if the genotype of any of our candidate aging genes can account for some of the variance in creatinine clearance, we added two terms to the model:
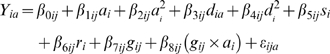
(5)In equation 5, *g_ij_* is the genotype of SNP *j* in subject *i*. We obtained estimates for three different inheritance models: additive, recessive and dominant. In the additive model *g* is 0, 1, or 2 for homozygous dominant, heterozygous, and homozygous recessive genotypes, respectively. In the recessive model, *g* is 0 for the homozygous dominant and heterozygous genotypes and *g* is 1 for the homozygous recessive genotype. In the dominant model, *g* is 0 for the homozygous dominant genotype and *g* is 1 for the heterozygous and homozygous recessive genotypes. For each SNP and each inheritance model, we compared the results from equation 5 to the results from equation 4 using a likelihood ratio test to generate a p-value for each SNP. Even though we included a self-reported race term in our models, we also confirmed the rs1711437 association with GFR by analyzing only the data points from Caucasian individuals (p = 0.0010).

For the InCHIANTI data, we used a simple linear regression model because the data are not longitudinal to search for SNP associations with creatinine clearance. We tested the three inheritance models for SNP association with creatinine clearance at every age (equation 6) and for SNP association with the rate of creatinine clearance decline with age (equation 7):
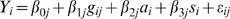
(6)

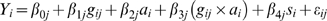
(7)In equations 6 and 7, *Y_i_* is the creatinine clearance of subject *i*, *g_ij_* is the genotype of subject *i* at SNP *j*, *a_i_* is the age of subject *i*, *s_i_* is the sex of subject *i*, and *ε_ij_* is a random error term. The coefficients were estimated by least squares from the data. In equation 6, our primary interest was *β*_1*j*_ values that significantly differed from zero, indicating that SNP *j* associates with creatinine clearance at every age. In equation 7, our primary interest was *β*_3*j*_ values that significantly differed from zero, indicating that SNP *j* associates with the rate of creatinine clearance decline with age.

### Testing for Evidence of SNP Association with GFR in Both Datasets

In order to be confident of a SNP association with GFR, we required the SNP to show evidence of association in both the BLSA and InCHIANTI populations. That is, we combined the p-values from the BLSA and InCHIANTI data using Fisher's method (equation 2) only if the individual p-values for a particular SNP and inheritance model in each population were both less than 0.05. We used the p-value from the likelihood ratio test for the BLSA data and the p-value from the *β*_1*j*_ estimate from equation 6 or the *β*_3*j*_ estimate from equation 7 for the InCHIANTI data to calculate the meta p-value.

### Permutation Analysis

To correct for multiple hypothesis testing, we performed permutations to test how often our results could appear by chance. We resampled the data for each population and each model 1000 times, keeping the genotypes together, but swapping the sample labels. The creatinine clearance, age, date and sex information remained together, but the 2011 SNP genotypes connected to each individual were changed in each permutation. Therefore, only the phenotype-genotype relationship was altered by permutation, the linkage disequilibrium patterns between SNPs remained the same. For each permutation, we calculated Fisher's meta p-values only when both individual p-values from each population were less than 0.05, as we did in the observed data. Then, for each model, we determined how many of the permutations met or exceeded the number of SNPs we found in the observed data at meta p-value thresholds. The permuted p-value was the number of permutations that met these criteria divided by 1000. Permuted p-values less than 0.05 were considered significant.

## Supporting Information

Table S1 SNPs tested for association with expression.
(0.13 MB XLS)

Table S2 Total expression associated SNPs.
(0.02 MB XLS)

Table S3 Allele-specific expression associated SNPs.
(0.04 MB XLS)

Table S4 SNPs tested for association with kidney aging.
(0.15 MB XLS)

Table S5 Common allele-specific expression across studies.
(0.02 MB XLS)
